# Relative Frequency of Primary Salivary Gland Tumors: Multicenter Study of 796 Cases from Riyadh, Saudi Arabia

**DOI:** 10.3390/medicina60122022

**Published:** 2024-12-08

**Authors:** Nasser AlMaden, Rawan AlYami, Ahmed Almotairi, Rasha Alrasheed, Bader Aldawasri, Mohammed Alwhabi, Assem Alrumeh, Nasser AlBishi, Abdullah Alqarni, Doaa Alghamdi, Asma Almazyad

**Affiliations:** 1 Dental Center, Prince Sultan Military Medical City, Riyadh 12233, Saudi Arabia; nmalmadan@moh.gov.sa; 2 Dental Specialist Center, Hafar Albatin 39921, Saudi Arabia; 3 King Abdulaziz Medical Center, National Guard Health Affairs, Riyadh 21423, Saudi Arabia; r00w.94@gmail.com; 4 King Abdulaziz International Medical Research Centre, Riyadh 22384, Saudi Arabia; 5 Department of Pathology, College of Medicine, King Saud University, Riyadh 11451, Saudi Arabia; aalmotairi1@ksa.edu.sa (A.A.); a.d.n-08@hotmail.com (A.A.); 6 Dental Hospital, King Saud University, Riyadh 11451, Saudi Arabia; ralrasheed2.c@ksu.edu.sa (R.A.); 434103961@student.ksu.edu.sa (B.A.); 7 Department of Pathology, Prince Sultan Military Medical City, Riyadh 12233, Saudi Arabia; mohammed.alwhabi@uhn.ca (M.A.); assem.alrumiah@uhn.ca (A.A.); 8 Department of Pathology, King Fahad Medical City, Riyadh 12231, Saudi Arabia; naoalbishi@moh.gov.sa (N.A.); dalghamdi@kfmc.med.sa (D.A.); 9 Maxillofacial Surgery and Diagnostic Science Department, College of Dentistry, King Saud bin Abdulaziz University for Health Sciences, Riyadh 11481, Saudi Arabia

**Keywords:** mesenchymal tumors, mucoepidermoid carcinoma, parotid tumors, pleomorphic adenoma, salivary gland tumors, tertiary hospitals, epidemiology

## Abstract

*Background and Objectives:* Salivary gland tumors (SGTs) are diverse lesions with varying morphological and clinical characteristics. Limited data exist on the distribution of SGTs in Saudi Arabia. We aimed to fill this gap by examining the distribution of SGTs across four tertiary hospitals in Riyadh. *Materials and Methods:* A retrospective analysis was conducted on SGT cases diagnosed from January 2010 to December 2022 to investigate the clinicopathological features (tumor location, patient sex, and age). Histological slides were evaluated by two independent certified oral pathologists and classified based on the latest 2022 World Health Organization classification (WHO). *Results:* There were 796 SGTs. Most cases occur in individuals in their 4th to 5th decades of life. The parotid gland was the predominant site (79.4%), followed by the submandibular gland (9.3%). More than half of the tumors (527, 66.2%) were benign, involving major and minor salivary glands. Pleomorphic adenoma was the most common benign tumor, accounting for 354 cases (44.5%), followed by Warthin tumor with 117 cases (14.7%). Mucoepidermoid carcinoma was the most prevalent malignant tumor, identified in 98 patients (12.3%). Additionally, 36 (4.5%) mesenchymal and 30 (3.8%) hematolymphoid non-epithelial SGTs were reported. *Conclusions:* This multicenter study is the largest of its kind in Saudi Arabia, identifying pleomorphic adenoma and mucoepidermoid carcinoma as the most commonly reported benign and malignant tumors, respectively. These findings offer valuable insights into the understanding of salivary gland tumors globally.

## 1. Introduction

Primary salivary gland tumors (SGTs) are rare lesions that exhibit remarkable diversity across organ systems. The global incidence of SGTs ranges from 0.4 to 13.5 cases per 100,000 individuals, with benign SGTs being more frequent than malignant ones, at a six-to-one ratio [1,2]. SGTs present with varying clinical behaviors and prognoses [3]. Advances in molecular techniques have led to ongoing revisions in SGT classification, introducing new entities and reclassifying existing ones [4,5,6,7,8].

Numerous studies have explored the global distribution of primary SGTs [9,10,11]. Pleomorphic adenoma (PA) is the most common benign SGT, while mucoepidermoid carcinoma (MEC) is the most frequent malignant type [12,13,14]. Adenoid cystic carcinoma (ACC) is the leading malignancy of minor salivary glands [15]. Most of these tumors occur in the parotid glands, with 77.7% being benign [16]. Intraoral SGTs are often found on the palatal mucosa, where benign cases range from 55.3% to 59.0% [12,15]. However, 90% of tongue-origin SGTs are malignant [16].

Research on SGTs in Saudi Arabia is limited. AlSalem et al. [17] conducted a retrospective study on malignant SGTs in major glands, utilizing data from the Saudi Cancer Registry and the International Classification of Disease for Oncology, 3rd Edition. However, this study focused exclusively on malignant epithelial entities and major salivary glands. Additionally, in 2022, an international collaboration examined the global distribution of SGTs, incorporating data from a single oral pathology laboratory in Riyadh [18]. While insightful, this study lacked detailed clinicopathological information specific to the Saudi population. Similarly, Alshiddi et al. [19] reported on SGTs from the same laboratory based on the 2005 WHO classification, which has since undergone significant revisions.

Notably, these studies concentrated on epithelial neoplasms, excluding the mesenchymal and hematolymphoid tumors of the salivary glands. Alsanie et al. [18] and Alshiddi et al. [19] used data from the same laboratory, potentially introducing biases toward oral cavity biopsies. Therefore, we aimed to provide an updated, comprehensive analysis of the relative frequency and the demographic distribution of primary benign and malignant SGTs, including epithelial, mesenchymal, and hematolymphoid entities, from four major tertiary hospitals in Riyadh, Saudi Arabia.

## 2. Methods

This retrospective study of SGTs was conducted from January 2010 to December 2022, based on data from four major tertiary hospitals in Riyadh, Saudi Arabia: Prince Sultan Military Medical City, King Fahad Medical City, King Abdulaziz Medical City, and King Khalid University Hospital. The study followed the Declaration of Helsinki and was approved by the review boards of the respective institutions.

A convenient sample of all cases of primary benign and malignant SGTs, as well as mesenchymal and hematolymphoid tumors in major and minor salivary glands, were identified. All relevant data, including patient age, sex, tumor location, and pathology report, were extrapolated from electronic medical records and tabulated in an Excel sheet. Data were stored in a secure electronic database with anonymized patient identities, accessible only to the authors. Informed consent was waived due to the retrospective nature of the study.

Histopathological slides were reviewed by two certified oral pathologists (A.A. and N.M.) and classified based on the 2022 World Health Organization (WHO) Classification. In cases of uncertainty, the two oral pathologists engaged in discussions until a consensus was reached. Cases for which consensus could not be achieved were excluded from the study.

Inclusion criteria of the study:

1. Cases with histopathological features consistent with SGT diagnosis.
2. Cases with available demographic and clinical information, pathology reports, and histology slides.

Exclusion criteria of the study:

1. Cases with incomplete data, duplicate records, and recurrences.
2. Cases diagnosed as metastatic tumors.
3. Tumors involving the skin, sinonasal region, or respiratory tract.

Descriptive analysis was performed for patient age (expressed decades), sex, SGT type, and tumor location (parotid, submandibular glands, sublingual glands, and minor salivary glands) using IBM SPSS Statistics for Windows, version 24.0 (IBM Corp., Armonk, NY, USA). Tumor-specific age was reported as mean (standard deviation), while sex, SGT type, and location data were presented as frequencies and percentages. Graphs were generated using STAT 14.2 software (StataCorp., College Station, TX, USA).

## 3. Results

**Figure 1:**
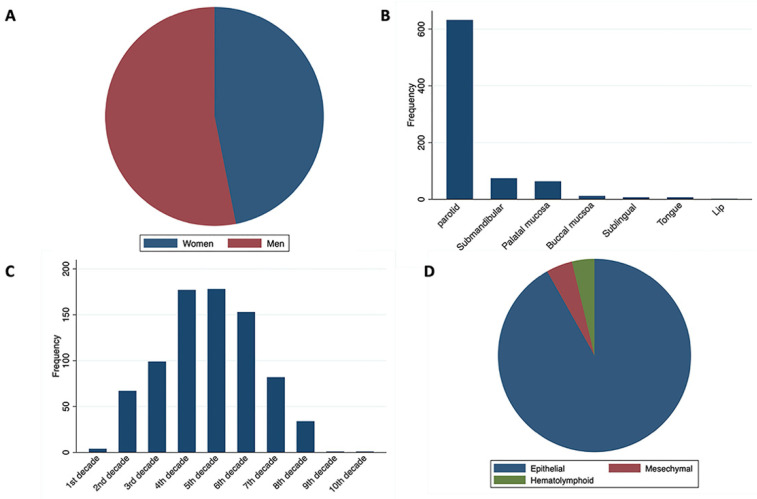
Demographic and topographic distribution of salivary gland tumors. (**A**) Sex distribution. (**B**) Location distribution. (**C**) Age (decade) distribution. (**D**) Salivary gland tumor distribution.

A total of 796 primary SGTs were retrieved from four tertiary hospital archives, with a mean age of 43.7 years (standard deviation [SD] 16.2). Of these, 355 (44.5%) patients were diagnosed in their 4th–5th decades. There was a slight male predominance (53.1%), with men having a higher mean age of 46.2 years (SD 15.6) compared to women, who had a mean age of 41 years (SD 16.5). The most common location was the parotid gland (79.4%), followed by submandibular glands (9.3%) and palatal mucosa (7.9%). Most tumors were epithelial (91.7%) (Figure 1).

### 3.1. Epithelial Neoplasms

**Figure 2:**
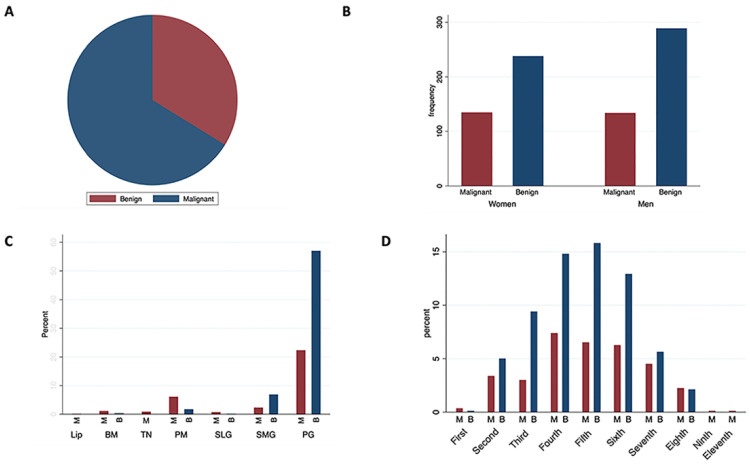
Demographic and clinical distribution of benign versus malignant tumors. (**A**) Overall distribution. (**B**) Sex distribution. (**C**) Location distribution. (**D**) Decade distribution.

More than half of the cases (495, 67.8%) were benign across both major and minor glands. The parotid and submandibular glands were the most common sites of benign tumors, with 454 of the 632 parotid cases and 55 of the 74 submandibular cases, respectively. In contrast, malignant neoplasms were more frequent in the sublingual and minor glands (Figure 2). The most common benign neoplasm was PA (354, 71.5%), followed by Warthin tumor (WT) (117, 23.6%) and basal cell adenoma (BCA) (9, 1.8%). PA and WT were mainly found in the parotid and submandibular glands, while BCA exclusively affected the parotid gland. Two cases of sclerosing polycystic adenoma were identified—one in the parotid and one in the submandibular gland.

The most common malignant neoplasm was MEC, accounting for 41.7% of malignant cases, with the parotid gland (55.1%) and palatal minor glands (27.5%) as frequent sites. ACC was the second most common malignancy (19.1%), mainly affecting the minor glands (48.9%) and parotid (24.4%). Acinic cell carcinoma (AcCC) occurred exclusively in the parotid gland. Thirteen cases of carcinoma ex PA were found, with affected patients being 15–19 years older than those with benign counterparts. Table 1 summarizes the demographic and topographic distribution of epithelial SGTs.

### 3.2. Mesenchymal Neoplasms

Thirty-six mesenchymal tumors were identified, of which 80.5% were benign. Sialolipoma was the most common benign tumor, with 15 cases (51.7%), including 12 in the parotid gland, with 60% occurring in men in their 3rd–5th decades. Five hemangiomas were reported, all in the parotid gland and predominantly in women (80%). Among malignant mesenchymal neoplasms, alveolar soft part sarcoma and synovial sarcoma were the most frequent, with two cases each (Table 2).

### 3.3. Hematolymphoid Conditions

Thirty hematolymphoid conditions were reported, most of which (86.7%) were lymphomas, with a diffuse large B-cell lymphoma (DLBCL) being the most common. There were also three cases of Kimura disease and one of myeloid sarcoma. Table 3 summarizes the demographic and topographic distribution of hematolymphoid conditions.

### 3.4. Pediatric Neoplasms

Sixty-four pediatric SGTs (8.1%) were identified. Most originated from epithelial glands, with PA accounting for 53.1%. MEC was the most common malignant pediatric tumor, with 17 cases (26.6%) located primarily in the parotid gland and palatal mucosa. AcCC was reported in three girls, all in the parotid gland. Two lymphoepithelial carcinomas (LECs) were also found in the parotid glands, one in a boy patient and one in a girl patient (Table 4).

## 4. Discussion

We analyzed 796 primary SGTs diagnosed between 2010 and 2022 at four major tertiary hospitals in Riyadh, Saudi Arabia. Unlike prior studies limited to single laboratories or focusing solely on malignant cases, this research provides a comprehensive view of both benign and malignant SGTs across various locations [17,19].

Our cohort showed a slight male predominance, aligning with studies from New Zealand and China [20,21]. However, other research noted a female predilection or equal sex distribution [18,22,23]. Benign tumors made up 65.6% of cases, consistent with the literature [20,24]. The regional variation in sex distribution may attributed to cultural and social behaviors influencing health-seeking practices, genetic or environmental factors, and potential disparities in study methodology and sample size. Most primary SGTs occurred between ages 40 and 70, peaking in the 4th and 5th decades. The percentage of benign tumors was slightly higher than previously reported [18,25], which may be influenced by Saudi Arabia’s younger population, as benign tumors are more prevalent in younger individuals [26]. Parotid and submandibular glands were frequently associated with benign tumors, while sublingual and minor salivary glands had higher malignancy rates, in line with previous studies [18,23].

### 4.1. Benign Epithelial Neoplasms

PA was the most common benign epithelial neoplasm (48.5%), followed by WT (16.0%), which was similar to previous findings [18,27]. PA exhibited a female predilection, with a mean age of 38.2 years (SD: 14.1), and most cases were diagnosed in the parotid and submandibular glands, peaking in the 4th–5th decades. This aligns with reports of a slight female predilection for PA [20,25]. However, our study showed a lower mean age than findings from the UK (47.4 years, SD: 17.5) [25] and Brazil (45 years) [28], although it was similar to Sri Lankan data (37.3 years) [29]. This trend reflects the younger demographics of Saudi Arabia, where 46.8% of the population falls within the 25–44 age range, emphasizing the influence of population structure on disease distribution.

Most WTs have been observed in the parotid gland, with none reported in the minor glands. In Germany, the incidence of WT is rising, and it is now recognized as the most common benign salivary gland neoplasm in the parotid gland [30,31]. However, our findings indicate that WT was the second most common neoplasm in our cohort. WT exhibited a clear man predominance with a ratio of 13.6:1, aligning with reports from China [16], contrasting with Europe, where the ratio is less pronounced (1.6:1–2.3:1) [25,30]. Similar results have been observed in New Zealand and the United States [32,33]. Historically, WT has been strongly associated with smoking, a habit commonly observed in men. Nowadays, in developed countries, both sexes smoke at relatively similar frequencies. However, in Saudi Arabia, men are more likely to smoke than women, with a ratio of 4:1 [34]. This disparity might explain the existing frequency of WT in men in our cohort.

We found no canalicular adenoma cases, unlike Alshiddi et al. [19], who reported two cases. This difference may stem from our focus on tertiary hospital samples instead of oral pathology lab specimens. We identified only one salivary gland neoplasm case on the lip, a typical site for canalicular adenoma.

### 4.2. Malignant Epithelial Neoplasms

MEC is the most commonly reported malignant SGT, primarily affecting the parotid gland, followed by minor glands, consistent with the existing literature. Some studies, however, report that minor glands are more common MEC sites, likely due to their oral pathology setting focus, whereas our cohort represents a tertiary hospital setting [16,18,25]. Most cases occurred in women with a woman-to-man ratio of 3:2. The mean age of female patients was 38 years, and male patients 43 years, which is lower than that of patients in the United States [35], Germany [36], and China [20].

ACC was the second most frequent epithelial SGT, with 45 cases (5.2%), mainly in minor glands, followed by the parotid gland, consistent with previous studies [18,20]. A female predilection was observed, with a ratio of 1.8:1, matching SEER data [37], although Tian’s reported an equal sex distribution [20]. AcCC, LEC, and salivary duct carcinoma showed high specificity for the parotid gland, which was consistent with global reports [16,20,38,39,40]. Polymorphous and cribriform adenocarcinomas were primarily seen in minor salivary glands, consistent with the existing literature [41,42,43]. Notably, we documented four adenocarcinoma NOS cases, including one that showed an intestinal-like morphology similar to previous reports [44,45].

### 4.3. Mesenchymal Neoplasm

Most mesenchymal neoplasms were benign (80.0%), with sialolipomas being the most common mesenchymal SGT, followed by hemangiomas. Torres et al. [46] reviewed 68 cases of mesenchymal SGTs and found that lipomas and hemangiomas were the most common, consistent with our findings. In contrast, a South Korean study identified schwannomas as the most common benign mesenchymal SGT, followed by lipomas [47]. However, other studies indicate lipomas and neurogenic tumors as more prevalent [48]. Interestingly, all the aforementioned papers documented cases of solitary fibrous tumors and plexiform neurofibromas, which were also observed in our cohort. Malignant mesenchymal SGTs were identified in seven patients, including two cases each of alveolar soft part sarcoma and synovial sarcoma, with 85.7% occurring in the parotid gland. Reports of malignant mesenchymal SGTs vary, with dermatofibrosarcoma protuberans, malignant peripheral nerve sheath tumors, embryonal rhabdomyosarcoma, synovial sarcoma, and leiomyosarcoma being the most commonly reported sarcomas in the parotid gland [47,48].

### 4.4. Hematolymphoid Conditions

Hematological conditions represented 3.7% of all cases, with DLBCL being the most common tumor, followed by MALT and follicular lymphomas. This distribution differs from previous reports, which indicated that MALT lymphoma was the most frequent hematolymphoid tumor, followed by DLBCL and follicular lymphoma [49,50,51]. Nodular lymphocyte-predominant Hodgkin lymphoma appeared four times, making it more prevalent than the classic type, which was consistent with Aggaimy [52]. We also encountered three cases of Kimura disease; a rare inflammatory condition most prevalent in Asia. Several cases have been reported in Saudi Arabia, with the salivary glands being frequently affected [53,54].

### 4.5. Pediatric Salivary Gland Tumors

Eight percent of SGTs in this cohort occurred in the pediatric population, with a slight predominance of benign diagnoses consistent with the literature [16]. However, a systematic review of 2937 cases indicated that 75.4% were malignant. MEC was the most frequently diagnosed tumor, accounting for 44.8% of the cases, followed by PA at 24.1% [55]. The most common benign and malignant SGTs in this cohort were PA and MEC, respectively, with AccC being the second most common malignant neoplasm. These findings align with data from Brazil, the United Kingdom, South Africa, and China [56,57]. Additionally, we reported two LEC cases, whereas Whaley et al. [58] reported one pediatric LEC case.

While our study included all SGTs diagnosed in four major tertiary hospitals in Riyadh to capture the majority of cases, its retrospective nature has certain limitations. These include incomplete data, which could introduce case selection bias and inconsistencies in data reporting, potentially affecting the quality and reliability of the results. Additionally, the multicenter design of the study poses a risk of data variability. To mitigate this, we thoroughly reviewed all cases histologically and ensured consensus in diagnosis and disease classification.

Our paper represents the first comprehensive study on primary SGTs in Saudi Arabia, offering a unique contribution by being the first report on mesenchymal tumors, with an additional emphasis on the pediatric population. Although SGTs are considered uncommon, they are clinically significant due to their complex diagnostic challenges. There is a growing international interest in the molecular pathways underlying SGTs, as evidenced by the increasing discovery of new entities and genetic profiles. This work serves as a crucial steppingstone for clinicians, pathologists, and researchers by providing insights into the epidemiology and characteristics of SGTs in the region. By shedding light on this area, our study encourages the further exploration of these tumors, particularly their molecular mechanisms, which are becoming more prominent in the modern treatment practice of head and neck cancers.

## 5. Conclusions

This retrospective study presents the most comprehensive analysis to date of the frequency and distribution of SGTs in Riyadh, Saudi Arabia, over the past 12 years. Our findings confirm and align with existing data, providing a foundational step toward advancing SGT research in Saudi Arabia. SGTs were primarily observed in the 4th to 5th decades of life, with a slight male predominance. The parotid gland was the most frequent site, accounting for nearly 80% of cases. Epithelial tumors constituted the majority (91.7%) of the reported cases, with PA and MEC being the most prevalent benign and malignant epithelial SGTs, respectively.

Mesenchymal tumors and hematolymphoid conditions were relatively rare, with sialolipoma and DLBCL being the most common subtypes in their respective categories. Pediatric SGTs accounted for 8.1% of the cases, showing a similar frequency distribution to adult epithelial SGTs. Future research should focus on the molecular characterization of these tumors, particularly adenocarcinoma, NOS, within the Saudi population. A deeper molecular understanding will enable clinicians to provide more tailored and effective care for patients with SGTs in Riyadh and other regions.

## Figures and Tables

**Table 1:** Distribution, demographic, and tomographic location of epithelial salivary gland neoplasm.

Neoplasm	No. (%)	Sex		Age Mean		Location				(%) of All Cases
Benign		Male	Female	Male	Female	Parotid	Submandibular	Sublingual	Minor SG	
Pleomorphic adenoma	354 (48.5%)	147 (41.5%)	207 (58.5%)	39 (SD: 12.6)	38 (SD: 15.1)	296 (82.6%)	42 (11.9%)	1 (0.3%)	15 (4.2%)	44.5%
Warthin tumor	117 (16.0%)	109 (93.2%)	8 (6.8%)	56 (SD: 9.6)	67 (SD: 12.8)	113 (96.6%)	4 (3.4%)	0	0	14.7%
Basal cell adenoma	9 (1.2%)	7 (77.8%)	2 (22.2%)	46 (SD: 13.4)	64 (SD: 11)	9 (100%)	0	0	0	1.1%
Myoepithelioma	6 (0.8%)	5 (83.3%)	1 (16.7%)	51 (SD: 30)	58	4 (66.7%)	1 (16.7%)	0	1 (16.7%)	0.7%
Oncocytoma	3 (0.4%)	2 (75.0%)	1 (25.0%)	57 (SD: 1.5)	74	2 (75.0%)	1 (25.0%)	0	0	0.4%
Cystadenoma	3 (0.4%)	2 (75.0%)	1 (25.0%)	49 (SD: 14.1)	49	3 (100%)	0	0	0	0.4%
Sclerosing polycystic adenoma	2 (0.3%)	1 (50.0%)	1 (50.0%)	20	72	1 (50.0%)	1 (50.0%)	0	0	0.3%
Sialadenoma papilliferum	1 (0.1%)	1 (100%)	0	32	NA	0	0	0	1 (100%)	0.1%
Total	495 (67.8%)	274 (55.4%)	221 (44.6%)			428 (86.5%)	49 (9.9%)	1 (0.2%)	17 (3.4%)	62.2%
***Malignant***										
Mucoepidermoid carcinoma	98 (13.4%)	41 (41.8%)	57 (58.2%)	43 (SD: 15.7)	38 (SD: 17.6)	64 (65.3%)	6 (6.1%)	1 (1.0%)	27 (27.6%)	12.3%
Adenoid cystic carcinoma	45 (6.2%)	16 (35.6%)	29 (64.4%)	48 (SD: 17.3)	46 (SD: 12.6)	11 (24.4%)	8 (17.8%)	4 (8.9%)	22 (48.9%)	5.7%
Acinic cell carcinoma	23 (3.2%)	9 (39.1%)	14 (60.9%)	44 (SD: 16.7)	41 (SD: 21)	23 (100%)	0	0	0	2.9%
Salivary duct carcinoma	14 (1.9%)	12 (85.7%)	2 (14.3%)	59 (SD: 9)	69 (SD: 8)	12 (85.7%)	2 (14.3%)	0	0	1.8%
Carcinoma ex pleomorphic adenoma **	13 (1.7%)	11 (84.6%)	2 (15.4%)	58 (SD: 10.5)	53 (SD: 18)	10 (76.9%)	2 (15.4%)	0	1 (7.7%)	1.6%
Polymorphous adenocarcinoma	10 (1.4%)	2 (20.0%)	8 (80.0%)	63 (SD: 23)	52 (SD: 11)	1 (10.0%)	0	0	9 (90.0%)	1.3%
Lymphoepithelial carcinoma	10 (1.4%)	8 (80.0%)	2 (20.0%)	38 (SD: 20.6)	43 (SD: 9.9)	10 (100%)	0	0	0	1.3%
Secretory carcinoma	5 (0.7%)	3 (60.0%)	2 (40.0%)	35 (SD: 23)	30 (SD: 1.4)	4 (80.0%)	0	0	1 (20.0%)	0.6%
Epithelial-myoepithelial carcinoma	5 (0.7%)	3 (60.0%)	2 (40.0%)	62 (SD: 10)	49 (SD: 5)	4 (80.0%)	0	0	1 (20.0%)	0.6%
Adenocarcinoma, NOS	4 (0.5%)	4 (100%)	0	65 (SD: 12.2)	NA	1 (0.25%)	0	1 (0.25%)	2 (50.0%)	0.5%
Cribriform adenocarcinoma	3 (0.4%)	1 (33.3%)	2 (66.7%	47	60 (SD: 1.4)	0	0	0	3 (100%)	0.3%
Sebaceous adenocarcinoma	1 (0.1%)	1 (100%)	0	51	NA	1 (100%)	0	0	0	0.1%
Myoepithelial carcinoma	1 (0.1%)	1 (100%)	0	40	NA	1 (100%)	0	0	0	0.1%
Large cell neuroendocrine carcinoma	1 (0.1%)	0	1 (100%)	NA	47	1 (100%)	0	0	0	0.1%
**Total**	235 (32.2%)	113 (48.0%)	122 (52.0%)			145 (61.7%)	18 (7.7%)	6 (2.5%)	66 (28.1%)	29.5%
**Grand total**	730 (100%)	387 (53.0%)	343 (47.0%)			573 (78.5%)	67 (9.2%)	7 (0.9%)	83 (11.4%)	91.7%

** including one carcinosarcoma ex PA.

**Table 2:** Distribution, demographic, and tomographic location of mesenchymal neoplasms.

Condition	No. (%)	Sex		Age Means		Location		(%) of All Cases
Benign		Male	Female	Male	Female	Parotid	Submandibular	
Sialolipoma	15 (51.8%)	9 (60.0%)	6 (40.0%)	47 (SD: 13.8)	40 (SD: 12.2)	12 (80.0%)	3 (20.0%)	1.9%
Hemangioma	5 (17.4%)	1 (20.0%)	4 (80.0%)	21	41 (SD: 21)	5 (100%)	0	0.6%
Schwannoma	2 (7.0%)	1 (50.0%)	1 (50.0%)	28	46	1 (50.0%)	1 (50.0%)	0.3%
Plexiform neurofibroma	1 (3.4%)	0	1 (100%)	NA	14	1 (100%)	0	0.1%
Pleomorphic lipoma	1 (3.4%)	0	1 (100%)	NA	56	0	1 (100%)	0.1%
Lymphangioma	1 (3.4%)	1 (100%)	0	29	NA	1 (100%)	0	0.1%
Epithelioid hemangioma	1 (3.4%)	0	1 (100%)	NA	48	1 (100%)	0	0.1%
Solitary fibrous tumor	1 (3.4%)	0	1 (100%)	NA	50	1 (100%)	0	0.1%
Intranodal palisaded myofibroblastoma	1 (3.4%)	1 (100%)	0	12	NA	1 (100%)	0	0.1%
Nodular fasciitis	1 (3.4%)	0	1 (100%)	NA	22	0	1 (100%)	0.1%
Total	29 (80.5%)	13 (44.8%)	16 (55.2%)			23 (79.3%)	6 (20.7%)	3.6%
Malignant								
Alveolar Soft part sarcoma	2 (28.6%)	2 (100%)	0	46 (SD: 23)	NA	1 (50.0%)	1 (50.0%)	0.3%
Synovial sarcoma	2 (28.6%)	1 (50.0%)	1 (50.0%)	58	31	2 (100%)	0	0.3%
Embryonal rhabdomyosarcoma	1 (14.3%)	1 (100%)	0	13	NA	1 (100%)	0	0.1%
Epithelioid angiosarcoma	1 (14.3%)	1 (100%)	0	35	NA	1 (100%)	0	0.1%
Liposarcoma	1 (14.3%)	0	1 (100%)	NA	59	1 (100%)	0	0.1%
Total	7 (19.5%)	5 (71.4%)	2 (28.6%)			6 (85.7%)	1 (14.3%)	0.9%

**Table 3:** Distribution, demographic, and tomographic location of hematolymphoid conditions.

Condition	No. (%)	Sex		Age Means		Location	(%) of All Cases
		Male	Female	Male	Female	Parotid	
Diffuse large B cell lymphoma	9 (30.0%)	7 (77.8%)	2 (22.2%)	52 (SD: 20)	42 (SD: 2)	9 (100%)	1.1%
MALT lymphoma	5 (16.7%)	1 (20.0%)	4 (80.0%)	77	50 (SD: 11)	5 (100%)	0.6%
Follicular lymphoma	5 (16.7%)	4 (80.0%)	1 (20.0%)	49 (SD: 20)	72	5 (100%)	0.6%
Hodgkin lymphoma (nodular lymphocyte predominant)	4 (13.3%)	3 (75.0%)	1 (25.0%)	25 (SD: 9.6)	50	4 (100%)	0.5%
Kimura disease	3 (10.0%)	2 (66.7%)	1 (33.3%)	52 (SD:17)	43	3 (100%)	0.4%
Hodgkin lymphoma (classic)	2 (6.7%)	1 (50.0%)	1 (50.0%)	47	73	2 (100%)	0.3%
Burkett lymphoma	1 (3.3%)	0	1 (100%)	N/A	2	1 (100%)	0.1%
Myeloid sarcoma	1 (3.3%)	0	1 (100%)	N/A	59	1 (100%)	0.1%
Total	30 (100%)	18 (60.0%)	12 (40.0%)			30 (100%)	3.7%

N/A; not applicable.

**Table 4:** Distribution, demographic, and tomographic location of pediatric salivary gland neoplasm.

Neoplasm	No. (%)	Sex		Location				
Epithelial Neoplasms		Male	Female	Parotid	Submandibular	Palatal Mucosa	Buccal Mucosa	Lip
Pleomorphic adenoma	34 (53.1%)	8 (23.5%)	26 (76.5%)	20 (58.8%)	8 (23.5%)	5 (14.7%)	1 (3.0%)	0
Mucoepidermoid carcinoma	17 (26.6%)	5 (29.4%)	12 (70.6%)	11 (64.7%)	1 (5.9%)	4 (23.5%)	0	1 (5.9%)
Acinic cell carcinoma	3 (4.7%)	0	3 (100%)	3 (100%)	0	0	0	0
Lymphoepithelial carcinoma	2 (3.1%)	1 (50%)	1 (50%)	2 (100%)	0	0	0	0
Adenoid cystic carcinoma	1 (1.6%)	1 (100%)	0	0	0	1 (100%)	0	0
Total	57 (89.0%)	15 (26.3%)	42 (73.7%)	36 (63.1%)	9 (15.8%)	10 (17.5%)	1 (1.8%)	1 (1.8%)
Mesenchymal neoplasms								
Hemangioma	1 (25.0%)	0	1 (100%)	1 (100%)	0	0	0	0
Intranodal palisaded myofibroblastoma	1 (25.0%)	1 (100%)	0	1 (100%)	0	0	0	0
Plexiform neurofibroma	1 (25.0%)	0	1 (100%)	1 (100%)	0	0	0	0
Embryonal rhabdomyosarcoma	1 (25.0%)	1 (100%)	0	1 (100%)	0	0	0	0
Total	4 (6.3%)	2 (50.0%)	2 (50.0%)	4 (100%)	0	0	0	0
Hematolymphoid neoplasms								
Diffuse large B cell lymphoma	1	1 (100%)	0	1	0	0	0	0
Burkett lymphoma	1	0	1 (100%)	1	0	0	0	0
Hodgkin lymphoma	1	1 (100%)	0	1	0	0	0	0
Total	3 (4.7%)	2 (66.7%)	1 (33.3%)	3 (100%)	0	0	0	0

## Data Availability

The data presented in this study are available on request from the corresponding author. The data are not publicly available due to hospital policies.
